# Adnexal Torsion with Dystrophic Calcifications in an Adolescent: A Chronic Entity?

**DOI:** 10.1155/2013/235459

**Published:** 2013-12-19

**Authors:** Pinar Solmaz Hasdemir, Fatma Eskicioglu, Gokhan Pekindil, Ali Riza Kandiloglu, Tevfik Guvenal

**Affiliations:** ^1^Department of Obstetrics and Gynecology, CBU School of Medicine, 45000 Manisa, Turkey; ^2^Department of Radiology, CBU School of Medicine, Manisa, Turkey; ^3^Department of Pathology, CBU School of Medicine, Manisa, Turkey

## Abstract

Intermittent pelvic pain caused by ovarian cysts in adolescence may be due to torsion or partial torsion of the ovary. We present a case of 18-year old adolescent with symptomatic left ovarian torsion with calcifications demonstrated by pelvic MRI and ultrasonography prior to surgery. The pathologic investigation demonstrated dystrophic calcifications. We speculated that the pattern of the intermittent pain in the story of the patient and the dystrophic calcifications in pathologic investigation which is thought that it might have been potentially developed as a result of chronic hypoxia due to intermittent partial torsions over a period of two years.

## 1. Introduction

Adnexal torsion should always be considered in the differential diagnosis of adnexal mass in an adolescent. We presented a chronic adnexal torsion case with calcifications. The pattern of the intermittent pain in the story of the patient and the dystrophic calcifications in pathologic investigation which is thought that it might have been potentially developed as a result of chronic hypoxia due to intermittent partial torsions over a period of two years.

## 2. Case

An 18-year-old girl presented to the outpatient clinic with an intermittent pelvic pain for the last two years with worsening pattern over the last 6-weeks. Her past medical history was unremarkable. Family history was remarkable for breast cancer in her mother. Physical examination revealed that there was a 10 cm soft mass in front of the uterus.

Laboratory findings were as follows: white blood cell count of 7.6 × 10^3^, serum hematocrit level of 26%, and serum Ca-125 level of 52.2 U/mL. Serum LDH, Alpha fetoprotein, CA 19-9, and beta-HCG levels were within normal limits. Pelvic MRI showed diffuse hypointensity of necrotic left ovary with small hyperintense peripheral area corresponding to hemorrhage and calcification at the periphery of left ovary as a hypointense focus. Pelvic ultrasonography with colour Doppler showed a 63∗56∗66 mm solid-cystic mass with no arterial perfusion.

**Figure 1:**
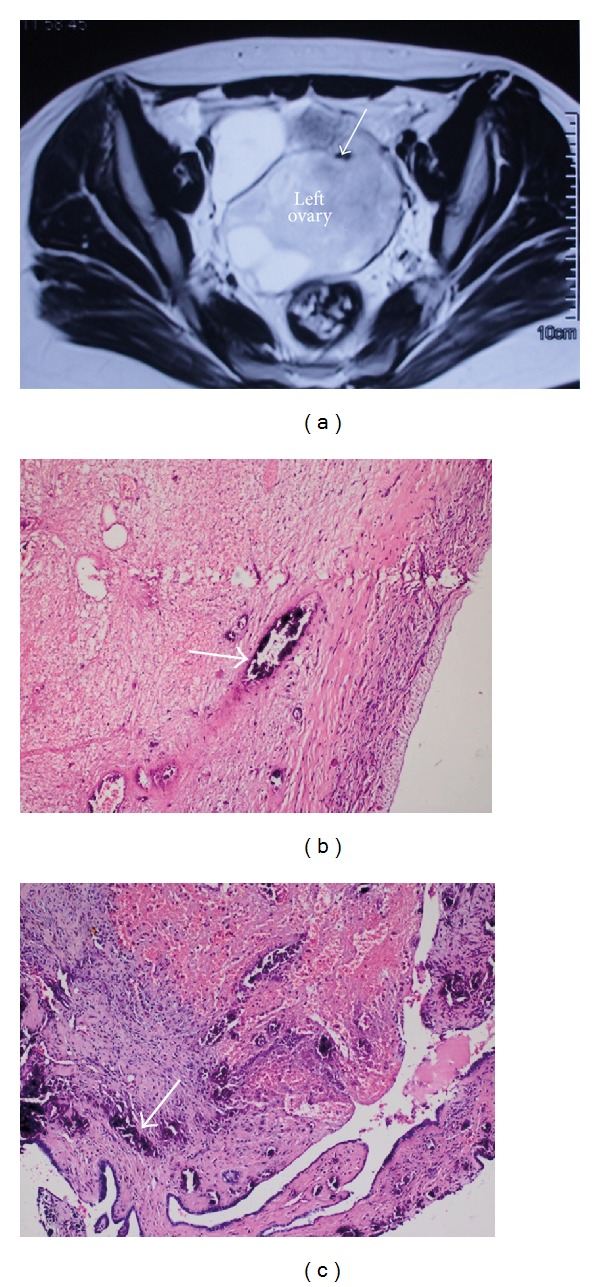
(a) Pelvic axial magnetic resonance imaging of the torsioned left ovary. T2w image showing the well-defined heterogeneous, hyperintense, enlarged left ovary along with a hypointense focus corresponding to the calcification (arrow) on the left upper peripheral border of enlarged ovary. (b) and (c) The microscopic views of dystrophic microcalcifications of the left ovary (b) and the left tuba uterina (c) stained with Hematoxilin-Eosin.

In the presence of the possibility of torsion, she was immediately taken to the operating room. On surgical exploration, the right ovary was seen in front of the uterus as a biloculated cystic mass (8 × 9 cm in diameter). Torsioned left adnexa (9.5 × 7 cm in diameter) was obliterated to Douglas pouch completely with adhesions. Cystectomy was made in the right ovary. The left adnexa were mobilized with blunt dissection. Both left tuba and ovary were seen three times tostioned in the presence of a cystic mass. Both of the tube and ovary were firm and full of coagulum. Total salphingo-oopherectomy was performed because of the diffuse tissue necrosis. The result of pathology from the right ovary was follicular cysts and corpus luteum, and from the left salphingo-oopherectomy material, was diffuse hemorrhagic necrosis and dystrophic calcifications in both ovary and tube (Figure 1).

## 3. Discussion

Clinical approach to the adnexal mass in adolescence differs from the adulthood counterparts in terms of the incidence of tumor types and the relative importance of the reproductive and endocrinological properties. The majority of adnexal masses in adolescents are asymptomatic and pathologically benign and often diagnosed as a result of complications like rupture or torsion. Adnexal torsion is reported to be the fifth most common gynecologic emergency condition encountered, with a prevalence of 2.7% [1]. Although it is most common in postmenarche period, it should be considered in any girl with an abdominal mass and any degree of abdominal pain [2].

Proper diagnosis is possible by using appropriate imaging modalities. Pelvic ultrasonography with color Doppler flow imaging is an excellent imaging technique especially in the evaluation of patients with acute pelvic pain [3].

Underlying ovarian tissue necrosis and type of surgery (complete removal of the ovary versus ovarian saving surgery) in these patients can be determined in part by the demonstration of calcifications by imaging modalities like ultrasonography and MRI [4].

We have limited knowledge about chronic torsion or intermittent partial torsions in pediatric and adolescent population. It has been demonstrated that partial torsion of the adnexa or the early phase of the torsion can cause massive ovarian edema [5].

Calcifications in cases with ovarian torsion have been rarely reported on either imaging modalities and/or pathologic examinations [6]. There are two types of well described pathological calcifications [7]. One of them is dystrophic calcification which is encountered in the necrotic areas whether they are of coagulative, caseous, or liquefactive type. The other pathologic calcification type is metastatic calcification which occurs in normal tissues whenever there is hypercalcemia. Dystrophic calcification defined as a type of calcification developing in the presence of chronic hypoxia and tissue necrosis has rarely been reported in these cases [8]. In our case, dystrophic calcification might have been potentially developed as result of chronic hypoxia due to intermittent partial torsions in a long time. These findings were consistent with the clinical history of the patient which was intermittent pain over 2 years. Intermittent pelvic pain with ovarian cysts in adolescent girls should be considered as adnexal torsion in differential diagnosis, especially if calcifications exist in imaging techniques.
